# Attitudes Toward Technology and Use of Fall Alert Wearables in Caregiving: Survey Study

**DOI:** 10.2196/23381

**Published:** 2021-01-27

**Authors:** Deborah Vollmer Dahlke, Shinduk Lee, Matthew Lee Smith, Tiffany Shubert, Stephen Popovich, Marcia G Ory

**Affiliations:** 1 Texas A&M Center for Population Health and Aging Texas A&M University College Station, TX United States; 2 DVD Associates, LLC Austin, TX United States; 3 Shubert Consulting Chapel Hill, NC United States; 4 Clairvoyant Networks Austin, TX United States

**Keywords:** wearables, falls alert technology, falls, caregivers, care recipients

## Abstract

**Background:**

Wearable technology for fall alerts among older adult care recipients is one of the more frequently studied areas of technology, given the concerning consequences of falls among this population. Falls are quite prevalent in later life. While there is a growing amount of literature on older adults’ acceptance of technology, less is known about how caregivers’ attitudes toward technology can impact care recipients’ use of such technology.

**Objective:**

The objective of our study was to examine associations between caregivers’ attitudes toward technology for caregiving and care recipients’ use of fall alert wearables.

**Methods:**

This study examined data collected with an online survey from 626 caregivers for adults 50 years and older. Adapted from the technology acceptance model, a structural equation model tested the following prespecified hypotheses: (1) higher perceived usefulness of technologies for caregiving would predict higher perceived value of and greater interest in technologies for caregiving; (2) higher perceived value of technologies for caregiving would predict greater interest in technologies for caregiving; and (3) greater interest in technologies for caregiving would predict greater use of fall alert wearables among care recipients. Additionally, we included demographic factors (eg, caregivers’ and care recipients’ ages) and caregiving context (eg, caregiver type and caregiving situation) as important predictors of care recipients’ use of fall alert wearables.

**Results:**

Of 626 total respondents, 548 (87.5%) with all valid responses were included in this study. Among care recipients, 28% used fall alert wearables. The final model had a good to fair model fit: a confirmatory factor index of 0.93, a standardized root mean square residual of 0.049, and root mean square error of approximation of 0.066. Caregivers’ perceived usefulness of technology was positively associated with their attitudes toward using technology in caregiving (b=.70, *P<*.001) and interest in using technology for caregiving (b=.22, *P*=.003). Greater perceived value of using technology in caregiving predicted greater interest in using technology for caregiving (b=.65, *P<*.001). Greater interest in using technology for caregiving was associated with greater likelihood of care recipients using fall alert wearables (b=.27, *P*<.001). The caregiver type had the strongest inverse relationship with care recipients’ use of fall alert wearables (unpaid vs paid caregiver) (b=–.33, *P*<.001).

**Conclusions:**

This study underscores the importance of caregivers’ attitudes in care recipients’ technology use for falls management. Raising awareness and improving perception about technologies for caregiving may help caregivers and care recipients adopt and better utilize technologies that can promote independence and enhance safety.

## Introduction

By 2035, adults 65 years and older in the United States are projected to outnumber children (under 18 years), mostly due to the continued aging of the Baby Boomer generation [1]. The proportion of older adults aged 65 years and older will increase from approximately 1 in 7 today to approximately 1 in 5 in 2030, when nearly all Baby Boomers will be of typical retirement age [2]. The majority of older adults will need long-term services and support during their lifetime [3]. The rapid growth of the oldest population (ie, those 85 years and older), individuals who tend to have more health conditions and disabilities, will compound the need for care with most requiring some level of care by either paid or unpaid caregivers [4].

According to the American Association of Retired Persons 2020 Report: Caregiving in America [4], caregivers report that the adults who receive care (care recipients) have more comorbid conditions that require care for medical and support than was reported by caregivers in 2015. Increasingly, unpaid caregivers are turning to assistive intelligent technology and wearables for assistance and support in caregiving [4]. Wearable technology is a category of electronic devices that are worn as accessories, embedded in clothing, implanted in the user's body, or even tattooed on the skin. Wearables can be powered by microprocessors to send and receive data via cellular networks and the internet [5-7]. Our review of recent literature on technology and caregiving offered multiple examples of digital technology adoption by caregivers and care recipients in the realms of education, care recipient data collection, sensors and monitoring, clinical care delivery, and social support [4,6,8-12]. While these studies [4,6,8-12] document the broad array of categories of digital and technology development, limited information is available about factors influencing care recipients’ technology adoption.

Wearable technology for fall alerts among older adult care recipients is one of the more frequently studied areas of technology, given the concerning consequences of falls among this population. Falls are quite prevalent in later life; approximately 1 in 4 community-dwelling older adults fall each year, and 20% of falls result in injury [13]. The consequences of falls can trigger a downward trajectory of dependence among older adults and can result in increased health care emergency room visits and hospitalization, staggering health care costs, and premature death [13-15]. Research suggests that caregivers are increasingly interested in purchasing and using wearable and other monitoring technology to help reduce caregiver burdens and allow older adults to remain independent in their own homes [9]. A recent literature review by Stavropoulos et al [10] included reviews of systematic reviews and case studies, including studies in which the aims were to assess if the caregiver was more comfortable due to the care recipient use of the wearable and if the care recipient felt more independent [10-12,16,17].

In recent years, falls have become viewed as preventable with evidence-based programs helping older adults prevent and better manage risk factors associated with falling [18]. Concurrently, technology tools are being developed to help older adults and their caregivers predict and prevent falls [18]. Of particular interest is the growing market for fall alert systems, which are intended to help older adults reduce fear of falling and stay independent by ensuring that help will be available in the event of a fall. There is now a plethora of medical alert systems with fall detection, and while there are market comparisons and a growing amount of literature on older adults’ acceptance of technology, less is known about how caregivers’ attitudes toward technology can impact care recipients’ use of such technology [6,8-12,16,17,19,20].

The objective of this paper is to better understand associations between caregivers’ attitudes toward technology for caregiving and care recipients’ use of fall alert wearables.

Based on an adapted framework of the Technology Acceptance Model (TAM) [21,22], we constructed a structural equation model to test the following hypotheses: (1) higher perceived usefulness of technologies for caregiving would predict higher perceived value of and greater interest in technologies for caregiving; (2) higher perceived value of technologies for caregiving would predict greater interest in technologies for caregiving; and (3) greater interest in technologies for caregiving would predict greater use of fall alert wearables among care recipients. 

We further based our analyses on specific demographic factors and caregiving contexts available from the data in our survey. In addition, our analyses were based on the following subhypotheses, supported in the literature: (1) younger age among caregivers would predict greater perceived usefulness, perceived value, and interest in using technology; (2) more demanding caregiving situations such as longer caregiving hours and dementia among care recipients would increase caregivers’ interest in technology; (3) older age among care recipients would predict greater health care needs and fall risks, hence more need for and use of fall alert–related technology [22,23]; and (4) use of or preference for using family (ie, unpaid) caregivers is most likely associated with economic status (ie, the ability to pay for caregivers) and the availability of unpaid caregivers as well as care recipients’ health conditions [23,24]. Correlates that predict the use of paid versus unpaid caregivers may also influence the use of fall alert technologies. Thus, we also examined whether care recipients’ use of fall alert wearables would be associated with caregiver type (paid or unpaid).

## Methods

### Model Construction

For this study, we adapted a validated model of technology acceptance by users in organizations, based on the TAM and an updated version (TAM2) [21,22], to guide the development of the survey instrument and data analyses to identify factors influencing caregivers’ and care recipients' use and perceptions of technologies associated with caregiving. A meta-analysis of 88 studies in different fields [25] indicated that TAM is “a powerful and robust predictive model” to understand technology acceptance of users in various contexts. Davis [26] originally empirically validated TAM to explain users’ willingness to use new technologies in organizations. In 2015, Marangunić and Granić [27] stated that TAM “has evolved to become the key model in understanding the predictors of human behavior toward potential acceptance or rejection of the technology.”

**Figure 1:**
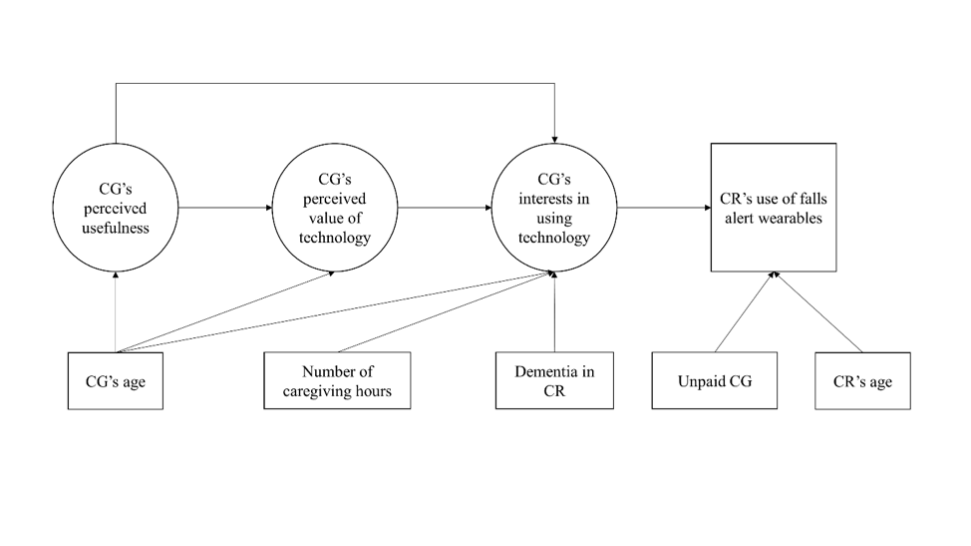
Initially hypothesized model predicting care recipient’s use of fall alert wearables. CG: caregiver; CR: care recipient.

In this study, we adapted TAM and TAM2 to build and test a framework (Figure 1) that includes factors regarding caregivers’ perceptions about how useful and valuable technologies might be in their caregiving activities. The key constructs of TAM and TAM2, perceived usefulness, attitudes toward using technology, intention to use, and usage behaviors were adapted to caregivers’ perceived usefulness, perceived value of technology, interests in using technology, and care recipients’ use of technology, respectively. Attitudes toward a behavior consists of personal evaluation of the specified behavior [27]. In the adapted model, attitudes toward using technology was adapted to perceived value of using technology in caregiving. Figure 1 illustrates our adaptation of the TAM2 model with its 3 key constructs (caregiver’s perceived usefulness, perceived value, and interest in using the technology) and other factors that potentially may directly or indirectly influence care recipients’ use of fall alert wearables.

### Data Source and Study Participants

This study used a cross-sectional online survey collected from 626 paid and unpaid caregivers for adults 50 years and older. The caregivers were recruited through an internet panel (Qualtrics XM) in November 2019. Survey respondents were eligible to be included in this study if they were aged 18 years or older, were either paid or unpaid, and provided at least 8 hours of care per week for at least one person who was over 50 years of age and who lived in a home environment. The recruited sample was targeted to resemble the population distribution across 4 US regions (eg, northwest: 17.2%; midwest: 20.9%; west: 23.8%; south: 38.1%) based on 2018 census data [28]. In addition to quotas by regions, quota sampling was predetermined for gender (approximately 75% female and 25% male), age (at least 50% of the sample 50 years and older), and race (maximum 60% White) to account for the known demographic characteristics of caregivers for middle-aged and older adults in the United States [4]. The survey design and study implementation were submitted to the Texas A&M University institutional review board and received approval for exemption (IRB2019-1128M).

### Variables

Caregiver’s perceived usefulness of technologies in caregiving was measured using 6 items on the extent technology helps with (1) reducing the caregiving burden in the future; (2) enabling the care recipient to live more independently; (3) enabling caregiver to have a better quality of life; (4) improving the caregiver’s relationship with their care recipient; (5) improving communication with the care recipient’s family and friends; and (6) improving communications with the care recipient’s health care team. Each item was measured on a 0-to-100-point slider, with higher scores indicating greater perceived usefulness. For the 6 items, Cronbach α=.92. The Kaiser-Meyer-Olkin measure was 0.89, and the Bartlett test of sphericity (χ^2^_15_=2458.77, *P*<.001) suggested that the data were appropriate for factor analysis. Exploratory factor analysis showed that the 6 items adequately loaded onto one construct (eg, scree plot and eigenvalues). Average variance extracted was 0.67 indicating that the construct sufficiently explains the item variances.

Caregiver’s attitudes toward various safety-related technology for caregiving was assessed by asking perceived value of (1) watches and wearables that enable emergency calls and provide easy to use communications with family members; (2) cameras and alerts to make the house safe; (3) wearable technology to track care recipient health conditions (eg, breathing, pulse, and blood pressure); (4) watches and wearable sensors to monitor and send emergency alerts about falls; (5) watches and sensors that provide care recipient's location; and (6) wearables and sensors that alert if care recipients are at risk for falls. The survey respondents rated perceived value of each technology on a 0-to-100-point slider, with higher scores indicating greater perceived value of the technology in caregiving. For the 6 items, Cronbach α was .91. The Kaiser-Meyer-Olkin was 0.90, and Bartlett test of sphericity was statistically significant (χ^2^_15_=2130.27, *P*<.001). Exploratory factor analysis showed that the 6 items adequately loaded onto one construct. The level of variance captured by the construct was considered acceptable with average variance extracted of 0.64.

Two items were used to measure caregiver’s interest in using technology for tracking their care recipient’s location and providing alerts if their care recipient is at risk for a fall. The valid response range for the 2 items was 0 to 100 points, using a slider with higher scores indicating greater interests in using the technology. The Spearman-Brown reliability estimate for the 2 items was 0.75.

The online survey collected sociodemographic characteristics of caregivers and caregiving context, as well as the caregiver's oldest care recipient’s age, dementia diagnosis status, and use of fall alert wearables (eg, pendant or other wearable to alert others that a fall has occurred). Sociodemographic characteristics of caregivers included age in years, gender, race/ethnicity, place of residence (rural vs urban), education (associate degree or less education vs bachelor degree or higher education), employment status (employed for wages or self-employed vs other), previous year’s household income (<US $50,000 vs ≥$50,000), and financial stress (ie, “In general, how do your finances usually work out at the end of the month? Do you find that you usually: end up with some money left over/have just enough money to make ends meet/not have enough money to make ends meet?”). Self-reported zip codes were approximated to the census tract–based rural-urban commuting area codes [29]. Caregiving-related information included caregiving type (informal or unpaid vs formal or paid) for the oldest care recipient and the number of weekly hours of caregiving for the oldest care recipient.

### Statistical Analyses

**Figure 2:**
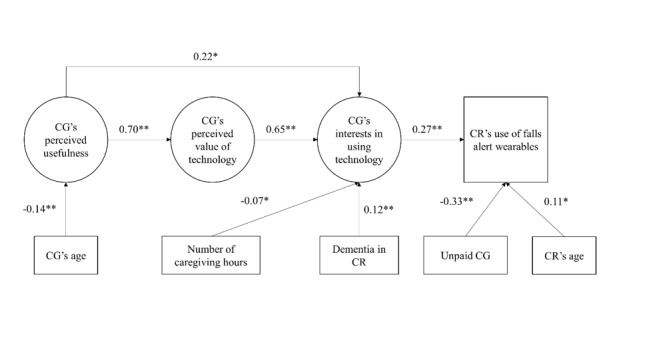
Revised model predicting care recipient’s use of falls alert wearables. CG: caregiver; CR: care recipient. *P<.05; **P<.001.

Characteristics of the study’s caregivers, their care recipients, and caregiving contexts, as well as caregivers’ attitudes toward using technology in caregiving, were described using mean and standard deviation or frequency and percentage. Independent group comparison (eg, 2-tailed independent *t* test or chi-square test) was used to compare each described characteristic by care recipient’s use of fall alert wearables. Next, a structural equation model was performed to test the hypothesized model (Figure 1). Goodness of fit was determined using confirmatory factor index (CFI), root mean square error of approximation (RMSEA), and standardized root mean square residual (SRMR)—good to fair was defined as CFI>0.90, RMSEA<0.08, and SRMR<0.08. Modification indices were also reviewed to explore potential model improvements. Figure 2 shows the final model used in this study. All statistical analyses were performed using SAS (version 9.4, SAS Institute) and with only included the caregivers who had valid data on all variables used in the structural equation model (548/626, 87.5%). Given potential differences between paid and unpaid caregivers, the independent group comparison was conducted to compare each described characteristic by caregivers’ paid status (Multimedia Appendix 1), and the hypothesized model (after excluding caregiver payment status) was tested separately among the paid (116/548, 21.2%) and unpaid (432/548, 78.8%) caregivers (Multimedia Appendix 2 and Multimedia Appendix 3).

## Results

### Study Population

Table 1 describes the characteristics of the caregivers and care recipients and the caregiving context. The mean age of the caregivers was 58.1 (SD 14.1) years, and the majority were females (417/547, 76.2%), non-Hispanic White (354/545, 65.0%), from an urban area (500/547, 91.4%) and had some college or higher educational attainment (420/548, 76.6%). Over 43% (237/548) were employed for wages or self-employed, and slightly more than half (279/548) had a total household income less than $50,000 in 2018. Approximately 55% (296/542) of caregivers reported some level of financial stress (ie, having just enough money to make ends meet or not having enough money to make ends meet). The mean age of care recipients was 74.5 (SD 11.93) years, and 23.4% (128/548) of caregivers reported their care recipient was diagnosed with dementia. The majority of the caregivers lived with the care recipient (311/548, 56.8%), were unpaid for the care or assistance they provided to their care recipients (432/548, 78.8%), and a reported weekly average of 37.5 (SD 28.98) hours providing care.

Fewer than 28% (153/548) of the study’s care recipients used a fall alert wearable. In a bivariate analyses comparing caregivers for those who do not use fall alert wearables to those who do use fall alert wearables found that the caregivers of those who used fall alert wearables were significantly younger (*P*<.001), less likely to be non-Hispanic White (*P*=.005), and under financial stress (*P*=.003). They were also more likely to be employed for wages or self-employed (*P<*.001). Furthermore, the care recipients who used fall alert wearables were significantly older (*P<*.001) and more likely to have dementia (*P*=.01) than those not using fall alert wearables. Caregivers of those who used fall alert wearables reported fewer weekly hours of caregiving (*P*=.002) and were also significantly less likely to be an unpaid caregiver (*P*<.001) or to live with the care recipient (*P*<.001). Caregivers of care recipients using fall alert wearables had significantly greater perceived usefulness (*P*<.001), perceived value (*P*<.001), and interest (*P*<.001) in using technology in caregiving than caregivers of those not using fall alert wearables.

**Table 1:** Characteristics of the study respondents and caregiving context and caregivers’ attitudes toward using technology in caregiving.

Characteristic			All (N=548)	Care recipients using fall alert wearables (n=153)	Care recipients not using fall alert wearables (n=395)	*P* value^a^
Age (years), mean (SD)			58.1 (14.07)	53.2 (16.58)	59.8 (12.90)	<.001
**Gender, n (%)**						.71
	Female		417 (76.2)	115 (75.2)	302 (76.6)	
	Male		131 (23.8)	38 (24.8)	93 (23.4)	
**Race/ethnicity, n (%)**						.005
	Non-Hispanic White		354 (65.0)	82 (53.6)	272 (69.4)	
	Non-Hispanic Black		93 (17.1)	38 (24.8)	55 (14.0)	
	Non-Hispanic Asian		35 (6.4)	10 (6.5)	25 (6.4)	
	Non-Hispanic other races		9 (1.7)	2 (1.3)	7 (1.8)	
	Hispanic		54 (9.9)	21 (13.7)	33 (8.4)	
**Education level, n (%)**						.87
	High school or lower		128 (23.4)	35 (22.9)	93 (23.5)	
	Some college or higher		420 (76.6)	118 (77.1)	302 (76.5)	
**Employment status, n (%)**						<.001
	Employed for wages or self-employed		237 (43.2)	96 (62.7)	141 (35.7)	
	Not employed for wages, not self-employed		311 (56.8)	57 (37.3)	254 (64.3)	
**Household income, n (%)**						.72
	Less than US $50,000		279 (50.9)	76 (49.7)	203 (51.4)	
	More than US $50,000		269 (49.1)	77 (50.3)	192 (48.6)	
**Financial stress, n (%)**						.003
	End up with some money left over		246 (45.4)	79 (52.3)	167 (42.7)	
	Have just enough money to make ends meet		212 (39.1)	61 (40.4)	151 (38.6)	
	Not have enough money to make ends meet		84 (15.5)	11 (7.3)	73 (18.7)	
**Residence, n (%)**						.29
	Rural		47 (8.6)	10 (6.5)	37 (9.4)	
	Urban		500 (91.4)	143 (93.5)	357 (90.6)	
**Care recipient**						
	Age (years), mean (SD)		74.5 (11.93)	77.2 (12.21)	73.5 (11.95)	<.001
	**Having dementia, n (%)**					.01
		Yes	128 (23.4)	47 (30.7)	81 (20.5)	
		No	420 (76.6)	106 (69.3)	314 (79.5)	
**Caregiving context**						
	**Paid for caregiving**					<.001
		Paid caregiver	116 (21.2)	68 (44.4)	48 (12.2)	
		Unpaid caregiver	432 (78.8)	85 (55.6)	347 (87.8)	
	Weekly hours of caregiving^b^, mean (SD)		37.5 (28.98)	31.3 (23.83)	39.3 (30.00)	.002
	**Living with the care recipient, n (%)**					<.001
		Yes	311 (56.8)	53 (34.6)	258 (65.3)	
		No	237 (43.2)	100 (65.4)	137 (34.7)	
**Caregivers’ attitudes^c^,** **mean (SD)**						
	Perceived usefulness		58.3 (25.57)	68.2 (21.94)	54.5 (25.86)	<.001
	Perceived value		63.5 (27.22)	73.6 (20.48)	59.5 (28.48)	<.001
	Interest		59.2 (30.40)	72.6 (26.02)	54.0 (30.40)	<.001

^a^Results from unadjusted independent group comparison between the group, in which care recipients use fall alert wearables, and another group, in which care recipients do not use fall alert wearables.

^b^Total weekly hours of caregiving was capped at 100 hours.

^c^Values ranged from 0 to 100, with a higher value indicating greater perceived usefulness, greater perceived value, or more interest in using technology in caregiving.

### Model Fit and Refinement

Goodness of fit, of the model shown in Figure 1, indicated good-to-fair model fit (CFI 0.93, SRMR 0.049, RMSEA 0.067). All hypothesized paths were statistically significant, except for the paths from age to perceived value (*P*=.73) and interests (*P*=.15) in technology in caregiving. Removing these 2 statistically insignificant paths did not change the direction or statistical significance of other paths in the model, and only minimal changes in the parameter estimates were observed, although the second model, shown in Figure 2, goodness-of-fit remained good-to-fair (CFI 0.93, SRMR 0.049, RMSEA 0.066).

### Path Coefficients

Figure 2 presents the standardized path coefficients of the final structural equation model. Caregivers’ perceived usefulness of technology was positively associated with their attitudes toward using technology in caregiving (b=.70, *P<*.001) and interests in using technology for caregiving (b=.22, *P*=.003). Greater perceived value of using technology in caregiving predicted greater interests in using technology for caregiving (b=.65, *P<*.001). Greater interests in using technology for caregiving was associated with greater likelihood of care recipients using fall alert wearables (b=.27, *P<*.001). Younger age of caregivers predicted greater perceived usefulness (b=–.14, *P*<.001). Care recipients of unpaid caregivers were less likely to use fall alert wearables (b=–.33, *P<*.001) than care recipients of paid caregivers. Fewer caregiving hours (b=–.07, *P=*.03) and presence of dementia among care recipients (b=.12, *P<*.001) predicted greater interests in using technology for caregiving. Care recipients’ age was positively associated with the use of fall alert wearables (b=.11, *P=*.004).

Table 2 presents direct and indirect effects of caregivers’ age and attitudes and caregiving context on care recipients’ use of fall alert wearables. In terms of total effects, caregivers’ interests in using technology for caregiving had the strongest positive effects on care recipients’ use of fall alert wearables (b=.27, *P*<.001), followed by caregivers’ perceived usefulness of technology in caregiving (b=.18, *P<*.001), and caregiver’s attitudes toward using technology in caregiving (b=.17, *P<*.001). The strongest inverse relationship was with caregiver type (unpaid vs paid caregiver) (b=–.33, *P<*.001). While the observed total effects were statistically significant, the magnitudes of the relationship tended to be weaker for hours of caregiving (b=–.02, *P=*.046), caregiver’s age (b=–.03*, P=*.003), and care recipient having dementia (b=.03, *P*<.001).

**Table 2:** Direct, indirect, and total effects of each predictor on care recipient’s use of fall alert wearables.

Variable	Direct effects		Indirect effects		Total effects	
	b^a^ (SE)	*P* value	b (SE)	*P* value	b (SE)	*P* value
Caregivers’ perceived usefulness of technology in caregiving	0	N/A^b^	.18 (0.03)	<.001	.18 (0.03)	<.001
Caregivers’ attitudes toward using technology in caregiving	0	N/A	.17 (0.03)	<.001	.17 (0.03)	<.001
Caregivers’ interests in using technology in caregiving	.27 (0.04)	<.001	0	N/A	.27 (0.04)	<.001
Caregivers’ age	0	N/A	–.03 (0.009)	.003	–.03 (0.009)	.003
Unpaid caregiver (vs paid caregiver)	–.33 (0.04)	<.001	0	N/A	–.33 (0.04)	<.001
Hours of caregiving	0	N/A	–.02 (0.01)	.046	–.02 (0.01)	.046
Care recipient having dementia	0	N/A	.03 (0.01)	<.001	.03 (0.01)	<.001
Care recipients’ age	.11 (0.04)	.004	0	N/A	.11 (0.04)	.004

^a^Standardized estimates.

^b^N/A: not applicable.

### Paid and Unpaid Caregivers

As shown in Table 1, nearly 79% (432/548) of the caregivers were unpaid. Multimedia Appendix 1 shows the comparison of caregiver and care recipient’s characteristics based on caregivers’ paid status. Compared to unpaid caregivers, paid caregivers were younger (48.0 years vs 60.8 years, *P*<.001); less likely to be non-Hispanic White (*P*<.001), having some college or higher educational attainment (*P*<.001), and living with the care recipients (*P*<.001); and more likely to be employed (*P*<.001). The oldest care recipients of paid caregivers were more likely to have dementia than the oldest care recipients of unpaid caregivers (*P*<0.001). There were statistically significant differences in the self-reported financial stress among paid and unpaid caregivers (*P*=.034). While there were 38.3% (44/115) and 49.6% (57/115) of paid caregivers having some money left over and having just enough money to make ends meet, respectively; there were 47.3% (202/427) and 36.3% (155/427) of unpaid caregivers having some money left over and having just enough money to make ends meet, respectively. Paid caregivers reported significantly greater perceived usefulness (*P*=.002) and interests (*P*=.004) in using technology in caregiving than caregivers of those not using fall alert wearables.

The model fit among paid caregiver was fair (CFI 0.93, SRMR 0.076, and RMSEA 0.062) and was comparable to the comprehensive model (CFI 0.93, SRMR 0.049, and RMSEA 0.066). The 3 prespecified hypotheses remained statistically significance, and corresponding path coefficients were comparable to the comprehensive model (Multimedia Appendix 2 shows the model among paid caregivers and Figure 2 shows the comprehensive model). Paid caregivers’ perceived usefulness of technology in caregiving was positively associated with perceived value of (b=.67, *P*<.001) and interest in (b=.36, *P*=.02) technology for caregiving. Higher perceived value of technology for caregiving was predicted greater interest in technologies for caregiving (b=.65, *P*<.001); and greater interest in technologies for caregiving predicted greater use of fall alert wearables among care recipients (b=.21, *P*=.02). None of the 4 subhypotheses remained statistically significant among paid caregivers.

The model fit among unpaid caregivers was good to fair (CFI 0.93, SRMR 0.051, and RMSEA 0.069) and was comparable to that of the comprehensive model (CFI 0.93, SRMR 0.049, and RMSEA 0.066). All path coefficients remained statistically significant, and path coefficients were comparable to the comprehensive model (Multimedia Appendix 3 shows the model among unpaid caregivers and Figure 2 shows the comprehensive model). Unpaid caregivers’ perceived usefulness of technology was positively associated with their attitudes toward using technology in caregiving (b=.71, *P<*.001) and interests in using technology for caregiving (b=.17, *P*=.03). Greater perceived value of using technology in caregiving predicted greater interests in using technology for caregiving (b=.67, *P<*.001). Greater interests in using technology for caregiving was associated with greater likelihood of care recipients using fall alert wearables (b=.31, *P<*.001). Younger age of unpaid caregivers predicted greater perceived usefulness (b=–.13, *P*=.005). Fewer caregiving hours (b=–.09, *P=*.02) and presence of dementia among care recipients (b=.13, *P<*.001) predicted greater interests in using technology for caregiving. Care recipients’ age was positively associated with the use of fall alert wearables (b=.11, *P=*.02).

For both paid and unpaid caregivers, caregiver’s interest in using technology for caregiving had the strongest positive effects on care recipient’s use of fall alert wearables (b=.21, *P*=.023 in paid caregivers; and b=.31, *P*=.028 in unpaid caregivers), followed by other attitudinal variables. Estimated total effects of caregiver’s perceived usefulness of technology in caregiving was b=.16 (*P*=.032) in paid caregivers and b=.20 (*P*<.001) in unpaid caregivers; and estimated total effects of caregiver’s attitudes toward using technology in caregiving was b=.13 (*P*=.036) in paid caregivers and b=.21 (*P*<.001) among unpaid caregivers.

## Discussion

### Principal Findings

From our analyses, we have demonstrated that the adapted TAM2 concepts of caregivers provide support for our hypotheses about care recipients’ use of fall alert wearables, which is reflective of previous literature [12,20-25,30]. Our model demonstrated that both high perceived usefulness and value of technology for caregiving was associated with greater interest in technologies for caregiving and that greater interest in technology for caregiving was predictive of greater use of fall alert wearables among care recipients, although only 28% (153/548) of our study’s care recipients used fall alerts. While statistically significant, our results suggested that younger age among caregivers was among the less powerful predictors of perceived use, attention and interest in technology for caregiving.

Our results demonstrated that the strongest predictor of care recipients’ use of fall alert wearable was the type of caregiver and that care recipients with paid caregivers were more likely to use this type of technology than care recipients with unpaid caregivers. While not expected, this may reflect the scenario where the path of caregiving for older adults typically begins with a family member or unpaid caregiver who lives in close proximity to the care recipient and provides human monitoring. Concerns for falls often results in investment in fall alert wearables for older adults living independently [9].

Our subhypothesis that more demanding caregiving situations, including longer hours of caregiving and instances of dementia among care recipients, was partially supported in this study. As hypothesized, dementia among care recipients positively predicts their use of fall alert wearables. However, contrary to our hypothesis, fewer caregiving hours was associated with care recipient’s use of fall alert wearables. A potential interpretation may be that caregivers providing fewer hours of care could be more inclined to use wearables to compensate for longer durations of nonsupervised time. According to the subgroup analyses based on caregiver’s payment status, the statistical significance of the subhypotheses are likely to be largely driven by unpaid care recipients, who constituted almost 79% (432/548) of the total analytic sample. While caregiver’s attitude toward technology in caregiving were significantly associated with care recipient’s use of fall alert wearables in both paid and unpaid caregivers, caregivers’ and care recipients’ age, and caregiving situations were significantly associated with care recipients’ use of fall alert wearables only among unpaid caregiver participants. The smaller sample size of paid caregivers may have limited the statistical power of the model. Another potential explanation is the differential involvement of paid and unpaid caregivers in caregiving decisions [29].

There is relatively little research that examines how caregivers and their care recipients (either paid or unpaid) actually use fall alert technology in their everyday lives or how such experiences may affect their safety and well-being. The little research that exists is limited in scale, often focused on care recipients with dementia and on cross-sectional interview methodologies focused on the adoption of the wearable fall alert technology [12,31-38]. Limited attention is typically given to how caregivers and their care recipients use wearable fall alert technology as their care and support needs change over time. In contrast, a study by van Heek et al [39] provided an empirical examination of caregivers’ acceptance of assistive technologies. However, van Heek et al [39] focused on design perspectives including gathering of data, data access, and storage duration, as well as perceived benefits and barriers, in order to integrate caregivers’ perspectives into design of technologies. Our results align with those of other recent studies [38-46] showing that there is a greater likelihood for adoption and use of fall alert wearables among care recipients with dementia, which is assumedly negotiated by the caregiver as a result of care recipient incapacity.

### Limitations

There were some limitations to our study. First, our caregiver population in the panel-based survey may not be representative of the caregiver population across the United States, despite our best efforts. While we have used quota sampling to match the distribution of key characteristics (eg, geographical region, age, gender, and race), this online sample excludes caregivers without access to internet and related technology (eg, computer, smartphone, or tablets). We assume that respondents were more willing to sign up to participate because they are comfortable with technology. Thus, caregivers who do have online access but are not as comfortable with technology may have elected not to participate. We also excluded caregivers who might have had online access but who had limited English proficiency. While we asked participants to self-identify as either paid or unpaid, there was no way to tell if there were subsets of unpaid caregivers who received some sort of stipend or benefit. With our cross-sectional design, it was not possible to draw conclusions about the causality between attitudes, caregiving contexts, and use of fall alert wearables. Additionally, the proposed model is limited by lack of potential factors, such as perceived ease of use for specific technology, fall history, and interpersonal relationships between caregivers and care recipients. In addition, the care recipients’ use of fall alert wearables were proxy-reported by caregivers, a further study using direct observation or self-reported measure by care recipients could supplement the proxy-reported evidence. More information on the types of technology and how the specific technologies are used would help establish circumstantial data to set out recommendations for practice and policy. Future research using in-depth interviews with caregivers to explore the nuances of technology adoption would be instructive for understanding more about the context driving our quantitative research findings. Despite these limitations, we believe our data and analyses provide important new information on how caregivers’ attitudes and values about technology influence adoption about the use of fall alert wearables for the protection and safety of their care recipients.

### Conclusion

With this study, we have taken a small step in addressing the knowledge gap about how caregiver attitudes affect adoption of assistive intelligent technology such as wearable fall alert technologies in caregiving, but much remains to be learned. With the growth of the aging population over the forthcoming years, and the anticipated rise of the occurrence of falls and related injuries based on the increasing numbers of older Americans, the caregiving workforce will benefit from advanced and effective technologies used in caregiving. It will continue to be crucial for public health researchers to keep pace with the advances of technology and maintain an advocacy role for both caretakers and care recipients in the adoption and use of technology to support their health and wellbeing.

## Appendix

Multimedia Appendix 1 Characteristics of the study respondents based on caregivers’ paid status.

Multimedia Appendix 2 Revised model predicting paid care recipient’s use of fall alert wearables.

Multimedia Appendix 3 Revised model predicting unpaid care recipient’s use of fall alert wearables.

## Abbreviations

CFI: confirmatory factor index
RMSEA: root mean square error of approximation
SRMR: standardized root mean square residual
TAM: Technology Acceptance Model
