# Supplementary material for: Attitudes Toward Technology and Use of Fall Alert Wearables in Caregiving: Survey Study
Source: JMIR Aging. 2021 Jan 27;4(1):e23381. doi: 10.2196/23381 (PMC8081189; doi:10.2196/23381)
Supplement: Multimedia Appendix 1 [file aging_v4i1e23381_app1.docx]

**Multimedia Appendix 1.**

**Characteristics of the study respondents and caregiving context and caregivers’ attitudes toward using technology in caregiving based on caregivers’ paid status**

| Characteristic | | All  (N=548) | Paid (n=116) | Unpaid (n=432) | *P* value^a^ |
| --- | --- | --- | --- | --- | --- |
| Age (years), mean (SD) | | 58.1 (14.07) | 48.0 (16.85) | 60.8 (11.85) | <.001 |
| **Gender, n (%)** | |  |  |  | .26 |
|  | Female | 417 (76.2) | 93 (80.2) | 324 (75.2) |  |
|  | Male | 131 (23.8) | 23 (19.8) | 107 (24.8) |  |
| **Race/ethnicity, n (%)** | |  |  |  | <.001 |
|  | Non-Hispanic White | 354 (65.0) | 56 (48.3) | 298 (69.5) |  |
|  | Non-Hispanic Black | 93 (17.1) | 33 (28.4) | 60 (14.0) |  |
|  | Non-Hispanic Asian | 35 (6.4) | 10 (8.6) | 25 (5.8) |  |
|  | Non-Hispanic other races | 9 (1.7) | 4 (3.4) | 5 (1.2) |  |
|  | Hispanic | 54 (9.9) | 13 (11.2) | 41 (9.6) |  |
| **Education level, n (%)** | |  |  |  | <.001 |
|  | High school or lower educational attainment | 128 (23.4) | 41 (35.3) | 87 (20.1) |  |
|  | Some college or higher educational attainment | 420 (76.6) | 75 (64.7) | 345 (79.9) |  |
| **Employment status, n (%)** | |  |  |  | <.001 |
|  | Employed for wages or self-employed | 237 (43.2) | 91 (78.4) | 146 (33.8) |  |
|  | Not employed for wages or self-employed | 311 (56.8) | 25 (21.6) | 286 (66.2) |  |
| **Household income, n (%)** | |  |  |  | .007 |
|  | Less than US $50,000 | 279 (50.9) | 72 (62.1) | 207 (47.9) |  |
|  | More than US $50,000 | 269 (49.1) | 44 (37.9) | 225 (52.1) |  |
| **Financial stress, n (%)** | |  |  |  | .03 |
|  | End up with some money left over | 246 (45.4) | 44 (38.3) | 202 (47.3) |  |
|  | Have just enough money to make ends meet | 212 (39.1) | 57 (49.6) | 155 (36.3) |  |
|  | Not have enough money to make ends meet | 84 (15.5) | 14 (12.2) | 70 (16.4) |  |
| **Residence, n (%)** | |  |  |  | .27 |
|  | Rural | 47 (8.6) | 7 (6.0) | 40 (9.3) |  |
|  | Urban | 500 (91.4) | 109 (94.0) | 391 (90.7) |  |
| **Care recipient** | |  |  |  |  |
|  | Age (years), mean (SD) | 74.5 (11.93) | 73.3 (13.04) | 74.9 (11.61) | .21 |
|  | Having dementia, n (%) |  |  |  | <.001 |
|  | Yes | 128 (23.4) | 48 (41.4) | 80 (18.5) |  |
|  | No | 420 (76.6) | 68 (58.6) | 352 (81.5) |  |
| **Caregiving context** | |  |  |  |  |
|  | Weekly hours of caregiving,^b^ mean (SD) | 37.5 (28.98) | 35.2 (20.6) | 38.1 (30.83) | .34 |
|  | Living with the care recipient, n (%) |  |  |  | <.001 |
|  | Yes | 311 (56.8) | 36 (31.0) | 275 (63.7) |  |
|  | No | 237 (43.2) | 80 (69.0) | 157 (36.3) |  |
| **Caregivers’ attitudes,^c^** **mean (SD)** | |  |  |  |  |
|  | Perceived usefulness | 58.3 (25.57) | 64.8 (23.29) | 56.6 (25.90) | .002 |
|  | Perceived value | 63.5 (27.22) | 66.8 (24.94) | 62.6 (27.75) | .140 |
|  | Interest | 59.2 (30.40) | 66.4 (27.88) | 57.2 (30.78) | .004 |
| **Care recipients using fall alert wearables, n (%)** | |  |  |  | <.001 |
|  | Yes | 153 (27.9) | 68 (58.6) | 85 (19.7) |  |
|  | No | 395 (72.1) | 48 (41.4) | 347 (80.3) |  |

^a^Results from unadjusted independent group comparison between the paid and unpaid caregivers.

^b^Total weekly hours of caregiving was capped at 100 hours.

^c^Values ranged from 0 to 100, with a higher value indicating greater perceived usefulness, greater perceived value, or more interest in using technology in caregiving.
