# Supplementary material for: Attitudes Toward Technology and Use of Fall Alert Wearables in Caregiving: Survey Study
Source: JMIR Aging. 2021 Jan 27;4(1):e23381. doi: 10.2196/23381 (PMC8081189; doi:10.2196/23381)
Supplement: Multimedia Appendix 2 [file aging_v4i1e23381_app2.docx]

**Multimedia Appendix 2.**

**Revised model predicting paid care recipient’s use of fall alert wearable. CG = Caregiver, CR = Care recipient, * *P* < .05, ** *P* < .001**

**
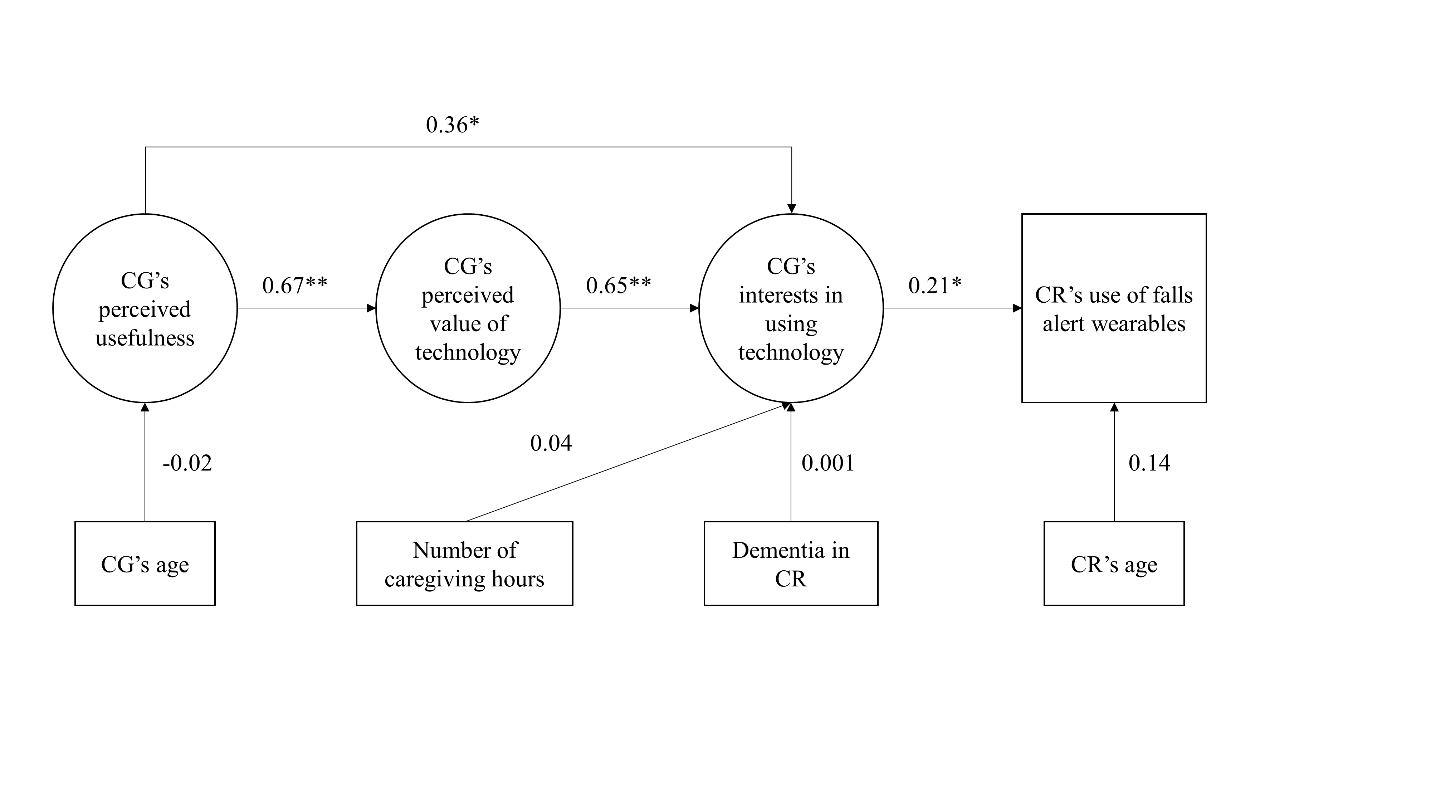
**
